# FOXO1 and FOXO3 Cooperatively Regulate Innate Lymphoid Cell Development

**DOI:** 10.3389/fimmu.2022.854312

**Published:** 2022-06-09

**Authors:** Thuy T. Luu, Jonas Nørskov Søndergaard, Lucía Peña-Pérez, Shabnam Kharazi, Aleksandra Krstic, Stephan Meinke, Laurent Schmied, Nicolai Frengen, Yaser Heshmati, Marcin Kierczak, Thibault Bouderlique, Arnika Kathleen Wagner, Charlotte Gustafsson, Benedict J. Chambers, Adnane Achour, Claudia Kutter, Petter Höglund, Robert Månsson, Nadir Kadri

**Affiliations:** ^1^ Department of Medicine Huddinge, Huddinge, Karolinska Institute, Stockholm, Sweden; ^2^ Center for Hematology and Regenerative Medicine, Huddinge, Karolinska Institute, Stockholm, Sweden; ^3^ Department of Microbiology, Tumor and Cell Biology, Science for Life Laboratory, Karolinska Institute, Stockholm, Sweden; ^4^ Department of Laboratory Medicine, Karolinska Institute, Stockholm, Sweden; ^5^ Department of Cell and Molecular Biology, National Bioinformatics Infrastructure Sweden, Science for Life Laboratory, Uppsala University, Uppsala, Sweden; ^6^ Science for Life Laboratory, Department of Medicine Solna, Karolinska Institute, and Division of Infectious Diseases, Karolinska University Hospital, Stockholm, Sweden; ^7^ Clinical Immunology and Transfusion Medicine, Karolinska University Hospital, Stockholm, Sweden; ^8^ Department of Hematology, Karolinska University Hospital, Stockholm, Sweden

**Keywords:** innate lymphocyte cells (ILCs), development, FOXO, natural killer cells, IL-15

## Abstract

Natural killer (NK) cells play roles in viral clearance and early surveillance against malignant transformation, yet our knowledge of the underlying mechanisms controlling their development and functions remain incomplete. To reveal cell fate-determining pathways in NK cell progenitors (NKP), we utilized an unbiased approach and generated comprehensive gene expression profiles of NK cell progenitors. We found that the NK cell program was gradually established in the CLP to preNKP and preNKP to rNKP transitions. In line with FOXO1 and FOXO3 being co-expressed through the NK developmental trajectory, the loss of both perturbed the establishment of the NK cell program and caused stalling in both NK cell development and maturation. In addition, we found that the combined loss of FOXO1 and FOXO3 caused specific changes to the composition of the non-cytotoxic innate lymphoid cell (ILC) subsets in bone marrow, spleen, and thymus. By combining transcriptome and chromatin profiling, we revealed that FOXO TFs ensure proper NK cell development at various lineage-commitment stages through orchestrating distinct molecular mechanisms. Combined FOXO1 and FOXO3 deficiency in common and innate lymphoid cell progenitors resulted in reduced expression of genes associated with NK cell development including ETS-1 and their downstream target genes. Lastly, we found that FOXO1 and FOXO3 controlled the survival of committed NK cells *via* gene regulation of IL-15Rβ (CD122) on rNKPs and bone marrow NK cells. Overall, we revealed that FOXO1 and FOXO3 function in a coordinated manner to regulate essential developmental genes at multiple stages during murine NK cell and ILC lineage commitment.

## Introduction

The evolutionarily conserved forkhead box transcription factors of the O class (FOXO) are critical regulators of metabolism, lifespan, fertility, proliferation, and cellular differentiation (1, 2). In mammals, the FOXO family is comprised of four members (FOXO1, FOXO3, FOXO4, and FOXO6) that, apart from FOXO6, are widely co-expressed throughout the immune system. Growth factor and survival signals activate the phosphoinositide 3-kinase-Akt signaling pathway, which leads to phosphorylation of the FOXOs and their subsequent nuclear exclusion and degradation (3). This counteracts the FOXO family’s role in promoting apoptosis (4, 5) and cell cycle arrest (6, 7). In the adaptive immune system, FOXOs control a wide range of functions including homing and survival of naïve T cells (1, 8, 9), expansion of CD8^+^ memory T cells (10, 11), differentiation of regulatory T cells (12–15), as well as B cell lineage commitment, homing, and germinal center proliferation (16–18).

NK cells are innate immune cells important for controlling viral infection and cancer (19–21). The IL-15-dependent NK cell lineage (22) is similar to B and T cells – derived from common lymphoid progenitors (CLP) (23). Downstream of the CLP, NK cells develop *via* two hierarchically related NK progenitor (NKP) stages originally defined by the loss of FMS tyrosine kinase 3 (FLT3) on preNKPs and the subsequent acquisition of IL-15Rβ (CD122) on refined NKPs (rNKP) (23). Recent studies have refined this developmental scheme by demonstrating that the preNKP compartment represents a heterogeneous population of innate lymphoid cell progenitors that give rise not only to NK cells but also to the non-cytotoxic ILC subsets (24–27).

Despite the identification of these intermediate NK cell progenitors and committed ILC progenitors within the preNKP compartment (24, 26), the precise stages where the ILC lineage-specific developmental programs are activated and the underlying mechanisms that lead to NK lineage restriction remain to be understood in detail. However, on the gene regulatory level, several transcription factors, including ETS1, NFIL3, and TCF7, have been shown to impact the development of NK cells at the preNKP and rNKP progenitor level (28–31).

Little is known about the role of the FOXOs in the development of non-cytotoxic ILCs. In contrast, their role in NK cell maturation has been investigated, but this has provided contradictory results. Relying on the specific Cre mediated deletion in Ncr1^+^ (NKp46) cells, Deng et al. observed a more mature phenotype in NK cells lacking FOXO1 or FOXO1 and FOXO3 (32). As this was not observed in NK cells lacking FOXO3 alone, this lead to the conclusion that FOXO1 is dispensable for NK cell development but negatively regulates NK cell maturation (32). Using a similar model, Wang et al., in contrast, observed that NK cell development was abrogated by the loss of FOXO1 (33). Noteworthy, Ncr1 expression is acquired only after commitment to the NK cell lineage and therefore, these studies did not address a potential role of the FOXOs in early NK cell development. In line with this, deletion of FOXO1 in the hematopoietic stem cell has been reported to result in increased frequencies of NK cell progenitors and committed NK cells (34). Together, this underlines the need for further studying the role of the FOXOs in NK cell progenitors and NK cell maturation.

Using ablation of FOXO1 and/or FOXO3 throughout the hematopoietic system, we show that the FOXOs are critical for NK progenitor development, establishment of the early NK gene regulatory network, and NK maturation. In addition, we show that the loss of FOXO perturbs the development of the non-cytotoxic ILC lineages. These findings provide novel insights into NK and ILC development and the gene regulatory program that underpins this process.

## Results

### The NK Gene Expression Program Is Initiated in preNKPs and rNKPs

**Figure 1:**
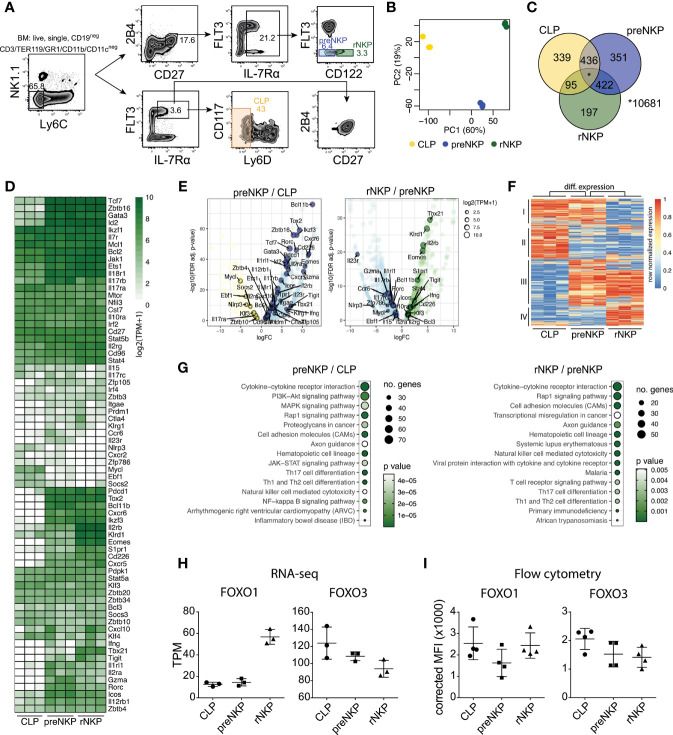
The rNKP gene expression program is established gradually in the CLP to preNKP and preNKP to rNKP developmental transitions. **(A)** Gating strategy for FACS sorting of BM NK cell progenitors (LY6D^neg^ CLP, preNKP and rNKP). **(B)** Principal component (PC) analysis of RNAseq data from indicated cell populations FACS sorted from WT (FOXO1^flox/flox^ FOXO3^flox/flox^) mice (n = 3 per population). The variation explained by each PC is displayed in parenthesis. **(C)** Venn diagram showing the overlap between expressed protein-coding genes in indicated populations. Genes with ≥0.3 transcript per million (TPM) in all three replicas were considered expressed. **(D)** Hierarchically clustered heatmaps showing expression of protein-coding genes important for NK cell or ILC development. **(E)** Volcano plots showing differentially expressed genes for the comparisons between preNKP versus CLP (left panel) and rNKP versus preNKP (right panel). Differentially expressed genes (regulated by ≥2-fold at an FDR<0.05) are highlighted in color. Circle sizes indicate expression values in log2(TPM+1). **(F)** Hierarchically clustered heatmap showing row normalized expression of differential expressed genes (identified in **E**). Clusters I-IV are indicated. **(G)** KEGG pathway analysis of differentially expressed genes comparing preNKP versus CLP (left) and rNKP versus preNKP (right). Genes regulated by ≥2-fold at an FDR<0.05 were considered differentially expressed and used in the analysis. The size and color of the circles indicate the number of genes in each category and significance of enrichment respectively. **(H–I)** Expression levels of FOXO1 and FOXO3 from indicated progenitor populations, obtained by **(H)** RNA-seq or **(I)** flow cytometry. Dots represent individual analysed animals (*n* = 2-4). Bars indicate mean and SD. Data shown in **(I)** is from one representative experiment out of two independent experiments.

To characterize gene regulation in early ILC and NK cell development, we performed RNA sequencing (RNA-seq) on FACS sorted LY6D^neg^ CLPs (35, 36), preNKPs and rNKPs (23–26) **(** **Figure 1A** and **Table S1** **)**. In agreement with preNKPs representing a developmental stage between CLPs and rNKPs (23), principal component analysis (PCA) revealed three distinct groups with the first component (PC1 60%) positioning the related progenitor subsets in the expected hierarchical order **(** **Figure 1B** **)**. Further in line with this, preNKPs expressed genes otherwise only expressed (≥0.3 TPM in all replicas) in CLPs or rNKPs **(** **Figure 1C** **)**.

Next, we investigated the expression pattern of genes known to be crucial for NK and ILC development (37, 38) **(** **Figure 1D** **)**. Many of these genes, including *Bcl11b, Tox2, Zbtb16, Tcf7, Rorc, Id2*, and *IL2rb*, were found to be upregulated at the preNKP stage **(** **Figure 1D** **)**. Looking directly at genes with significant expression changes in the CLP to preNKP **(** **Figure 1E** left) and preNKP to rNKP **(** **Figure 1E** right) transitions, revealed an overall pattern where preNKPs displayed down-regulation of genes expressed at the CLP stage (**Figure 1F**, cluster II, and in part I) and up-regulate genes expressed at the rNKP stage **(** **Figure 1F**, cluster III, and in part IV). When annotated (using Metascape), we as expected found that cluster I and II were enriched for B cell lineage associated genes (35, 36). In contrast, genes in cluster III and IV were enriched for genes associated with the NK cell lineage **(** **Figure S1A** **)**. Hence, suggesting that the B-lineage associated gene program observed in CLPs (35, 36) is shut down in preNKPs. The preNKPs instead adopt a general ILC gene program before a more refined NK gene program is established in the NK-lineage committed rNKPs (23).

To characterize changes in cell-signaling pathways occurring at the developmental transitions, we performed KEGG pathway analysis (**Figure 1G**) on the differentially expressed genes (**Figures 1E, F**). This revealed a significant enrichment of genes involved in cytokine-cytokine receptor interactions as well as in the PI3K-Akt, MAPK, and Rap1 signaling pathways **(** **Figure 1G** **)**. This is in line with prior observations of the critical involvement of cytokines and downstream signaling for early NK cell development and maturation (22, 39–41). Interestingly, the cytokine, PI3K-Akt, MAPK, and Rap1 pathways all coalesced on the FOXO family. This by either modulating FOXO localization and activity or by altering expression of genes that are direct transcriptional targets of the FOXO family (42–45). We found that FOXO1 and FOXO3 were co-expressed in LY6D^neg^ CLP, preNKP, and rNKP on the mRNA level **(** **Figure 1H** **)**. To further validate this observation, we confirmed that FOXO1 and FOXO3 were expressed at the protein level at all three progenitor stags **(** **Figure 1I** **)** as well as in committed NK cells from spleen and BM **(** **Figures S1B, C** **)**. This prompted us to further explore the role of the FOXO family in NK cell development (32, 34).

### NK Cell Development and Maturation Are Dependent on the FOXO Proteins

To understand the role of FOXO1 and FOXO3 in NK cell development, we utilized Vav-iCre (46) to conditionally ablate FOXO1 (FOXO1^ΔVav^) (47) and FOXO3 (FOXO3^ΔVav^) (48) individually or in combination (FOXO1,3^ΔVav^) throughout the hematopoietic system. Littermates lacking Vav-iCre (mainly FOXO1^flox/flox^FOXO3^flox/flox^ animals) were utilized as controls (WT). While residual FOXO1 and FOXO3 proteins were detected in the conditional mice (**Figure S2A**), the floxed DNA binding domains of both genes were found to be very efficiently deleted by Vav-iCre when investigated at the mRNA level **(** **Figure S2B** **)**.

**Figure 2:**
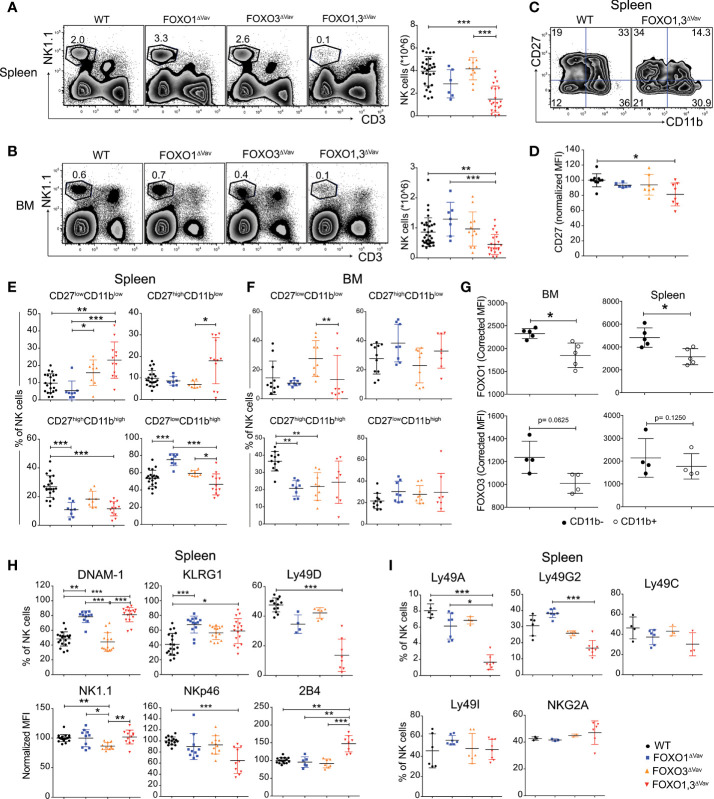
NK cell development is dependent on FOXO1 and FOXO3. **(A)** Representative flow cytometry profiles (left panel) and total number of NK cells (single, live NK1.1^+^CD3^-^ cells) (right panel) in spleen from animals with the indicated genotypes (n = 6-14). **(B)** Representative flow cytometry profiles (left panel) and total number of NK cells (single, live NK1.1^+^CD3^-^ cells) in bone marrow (BM) (right panel) from animals with the indicated genotypes (n=7-17). **(C)** Representative flow cytometry profiles showing splenic NK cell maturation stages in animals with the indicated genotypes. **(D)** CD27 MFI of CD27^+^ NK cells from spleens (n = 6-12). **(E)** Frequency (%) of splenic NK cells from each indicated maturation stage (*n* = 7-17). **(F)** Frequency (%) of BM NK cells from each indicated maturation stage (*n* = 8-11). **(G)** FOXO1 and FOXO3 protein expression (MFI) in BM and splenic NK cells (n = 4-5) from WT mice. Data is from one representative experiment out of two independent experiments. **(H)** Frequency (%) or normalized MFI of indicated activating receptors on splenic NK cells (*n* = 14-19). **(I)** Frequency (%) of splenic NK cells with indicated inhibitory receptors (*n* = 3-8). In panels **(A, B, D–I)**: dots represent individual analyzed animals; bars indicate mean and SD; *, ** and *** indicates p-values <0.05, <0.01 and <0.001 respectively. P-values were calculated using: Kruskal Wallis tests with Dunn’s multiple comparisons test (panels **A, B, D–F, H–I**) or the paired non-parametric Wilcoxon T test (panel G). Symbols utilized to indicate the genotype of analyzed mice throughout the panels are shown in the bottom right corner of the figure.

Neither the loss of FOXO1 or FOXO3 alone resulted in a significant alteration to the number of NK cells in spleen and BM **(** **Figures 2A, B** **)**. In sharp contrast, NK cell numbers were severely reduced in the spleen (5-10-fold reduced) and clearly decreased in the BM of FOXO1,3^ΔVav^ animals (2-5-fold reduced) **(** **Figures 2A, B** **)**. Altogether, this implies that FOXO1 and FOXO3 display functional redundancy in NK cell development and together are critical for the generation or maintenance of normal NK cell numbers.

We next assessed NK cell maturation in the three FOXO-deficient mouse strains based on CD11b and CD27 expression (49, 50). In this scheme, the first stage of NK cell maturation is characterized by low expression of both CD27 and CD11b (CD27^low^CD11b^low^). CD27 is then increased (CD27^high^CD11b^low^) before the subsequent upregulation of CD11b (CD27^high^CD11b^high^) and finally CD27 being down-regulated again (CD27^low^CD11b^high^) at the last step of maturation. The expression of CD27 on CD27^high^ NK cells was significantly reduced in FOXO1,3^ΔVav^ as compared to WT **(** **Figures 2C, D** **)** but regardless, all four maturation stages could be distinguished **(** **Figure 2C** **)**. Analysis of the maturation subsets in spleen revealed that FOXO1,3^ΔVav^ NK cells were less mature compared to NK cells from WT and single knockouts, with an accumulation of the CD27^low^CD11b^low^ population and a significant increase of the CD27^high^CD11b^low^ population **(** **Figure 2E** **)**. Interestingly, there was an accumulation of the terminally differentiated mature CD27^low^CD11b^high^ NK cells in FOXO1^ΔVav^ mice, that was not observed in FOXO3^ΔVav^ and FOXO1,3^ΔVav^ mice **(** **Figure 2E** **)**. This suggests that in the periphery FOXO1 might act as a brake for FOXO3-driven maturation as its absence only has an effect when FOXO3 is present. In contrast to what was found in the spleen, we observed no accumulation of the immature CD27^low^CD11b^low^ subset and only a trend towards mature CD27^high^CD11b^high^ NK cells being reduced in the BM of FOXO1,3^ΔVav^ mice **(** **Figure 2F** **)**.

To investigate whether the observed phenotype had any relation to FOXO expression, we quantified FOXO protein expression in early (CD11b^low^) and late (CD11b^high^) NK maturation **(** **Figure 2G** **)** in WT mice. In line with the reduced generation of later CD11b^high^ NK cells in FOXO1,3^ΔVav^ mice, we found that both FOXO1 and FOXO3 generally displayed higher expression in the more immature CD11b^low^ NK fraction **(** **Figure 2G** **)**. Together this suggests that the FOXO proteins to a higher extent influence developmental progression of CD11b^low^ NK cells and that peripheral NK cell development is more dependent on the FOXO proteins than BM NK cell development. The latter in line with the higher FOXO protein expression observed in splenic NK cells (**Figures S1B, C**).

### Loss of FOXO Results in Perturbed NK Receptor Expression

We next investigated the impact of the FOXO genes on the expression of activating and inhibitory receptors that control signaling and functionality in NK cells (21, 51). Loss of FOXO3 caused no significant changes in the NK receptor repertoire except for a reduction in NK1.1 expression **(** **Figures 2H, I** and **Figure S3A, B** **)**. In contrast, the loss of FOXO1 alone was enough to cause significant changes in DNAM-1 and KLRG1 expression **(** **Figure 2H** **)**. As FOXO1,3^ΔVav^ mice displayed a similar phenotype as FOXO1^ΔVav^ mice **(** **Figure 2H** **),** this suggests that the perturbation of DNAM-1 and KLRG1 is caused solely by the loss of FOXO1. In addition, FOXO1,3^ΔVav^ mice uniquely displayed significant changes in the expression of the activating receptors Ly49D, NKp46 and 2B4 **(** **Figure 2H** and **Figure S5A** **)** as well as the inhibitory receptors Ly49A and Ly49G2 **(** **Figure 2I** and **Figure S5B** **)**. Hence, the FOXO transcription factors individually or cooperatively influence the expression of specific activating and inhibitory receptors.

### CD122 Expression on NK Cells Is Reduced in the Absence of FOXO1 and FOXO3

**Figure 3:**
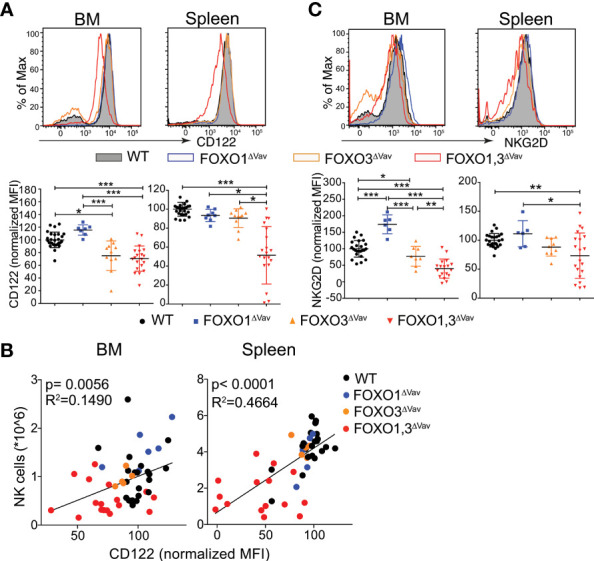
FOXO regulates CD122 expression on NK cells. **(A)** Representative flow cytometry profiles (top) and normalized MFI (bottom panel) showing CD122 expression on BM and splenic NK cells (n = 8-28). **(B)** Correlation between CD122 expression and NK cell numbers (n = 50). **(C)** Representative flow cytometry profiles (top) and normalized MFI (bottom panel) showing NKG2D expression on BM and splenic NK cells (n = 6-26). In panels **(A–C)**: dots represent individual analyzed animals; bars indicate mean and SD; *, ** and *** indicates p-values <0.05, <0.01 and <0.001 respectively. P-values were calculated using: Kruskal Wallis tests with Dunn’s multiple comparisons test (panels **A, C**) and linear regression (panel **B**).

As IL-15 is an important cytokine for NK cell development and survival (52), we investigated if the reduced expression of CD122 - a central component of the IL-15 receptor on NK cells (38) - could be a contributing factor to the observed reduction in NK cell numbers in FOXO1,3^ΔVav^ mice. We found a significant reduction in CD122 expression on both BM and splenic NK cells from FOXO1,3^ΔVav^ mice **(** **Figure 3A** **)** and that there was a clear correlation between NK cell numbers and CD122 expression in peripheral NK cells **(** **Figure 3B** **)**. Interestingly, dependence on the FOXO proteins differed between BM and splenic NK cells with the CD122 expression on BM NK cells seemingly only relying on FOXO3 **(** **Figure 3A** **)**. In contrast, the combined deletion was required to affect CD122 expression in splenic NK cells (**Figure 3A**). This, potentially due to the higher expression of FOXO proteins in splenic NK cells as compared to BM NK cells **(** **Figures S1B, C** **)**.

It has previously been shown that the expression of the activating receptor NKG2D on NK cells is dependent on IL-15 signaling (53–55) and hence it can be utilized as a surrogate marker for IL-15 signaling. Much resembling the expression pattern of CD122 (**Figure 3A**), we found that NKG2D expression was significantly reduced on both BM and splenic NK ells from FOXO1,3^ΔVav^ mice but that the dependence on the FOXO proteins varied between BM and spleen (**Figure 3C**). Hence, we concluded that the loss of FOXO results in reduced surface expression of CD122 and decreased IL-15 signaling. This strongly suggests that the reduced NK cell numbers in part can be directly contributed to a diminished IL-15 response in FOXO1,3^ΔVav^ NK cells.

### FOXO Deficiency Results in a Developmental Block at the preNKP to rNKP Transition

**Figure 4:**
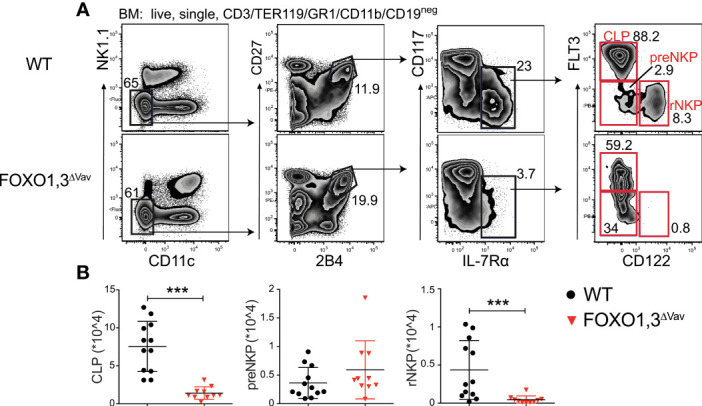
FOXO deficiency results in a block at the preNKP to rNKP transition. **(A)** Representative flow cytometry profiles showing the identification of CLP, preNKP and rNKP in animals with the indicated genotype. **(B)** Total number of CLP, preNKP and rNKP in BM of animals with the indicated genotype (*n* = 10-12). In panel **(B)**: dots represent individual analyzed animals; p-values were calculated using Mann-Whitney; bars indicate mean and SD; *** indicates p-values <0.001

The significant decrease in NK cell numbers (**Figure 2B**) coupled with the mild phenotype in BM NK cell maturation (**Figure 2F**) in FOXO1,3^ΔVav^ mice, hinted at a defect in NK cell progenitors. To investigate this, we performed phenotypic analysis of the CLP, preNKP, and rNKP compartments **(** **Figure 4A** **)**. This showed a significant decrease in the numbers of CLPs in FOXO1,3^ΔVav^ mice but no overt changes to the number of preNKPs **(** **Figures 4A, B** **)**. In contrast, the downstream CD122^+^ (IL15Rβ) rNKP population was significantly decreased in the FOXO1,3^ΔVav^ mice (**Figures 4A, B**). No significant changes were observed in preNKP and rNKP numbers in mice lacking only FOXO1 or FOXO3 alone (**Figure S4**). Hence, the combined loss of FOXO1 and FOXO3 results in seemingly increased generation of preNKPs from CLPs and developmental block at the preNKP to rNKP transition. The latter argues for a significant part of the observed reduction in NK cell numbers – particularly in BM – being due to decreased generation of early NK cell progenitors.

### The Perturbation in NK Development Is Intrinsic to the Hematopoietic Cells

To verify that the observed phenotype was intrinsic to hematopoiesis, we utilized the CD45.1/2 system and adoptively transfered FOXO1,3^ΔVav^ BM (CD45.2) to irradiated WT (CD45.1) hosts. The number of NK cells generated 12 weeks post transplantation from adoptively transferred FOXO1,3^ΔVav^ BM cells were markedly reduced in spleen **(** **Figures S5A, B** left**)**, BM **(** **Figure S5B** middle**)**, and blood (**Figure S5B** right**)**. The perturbation in NK maturation was also recapitulated with reconstituted FOXO1,3^ΔVav^ NK cells displaying accumulation of immature (CD27^low^CD11b^low^) NK cells and reduced number of more mature (CD27^high^CD11b^high^) NK cells (**Figure S5C**). Further, we observed a significant reduction in CLP numbers and block at the preNKP to rNKP transition in the progeny of FOXO1,3^ΔVav^ BM cells **(** **Figures S5D, E** **)**. Hence, the presence of normal cells in the transplantation setting does not rescue NK cell development from FOXO1,3^ΔVav^ donor BM cells, supporting the notion of a cell autonomous FOXO requirement in the regulation of early NK progenitors and NK cell maturation.

### Loss of FOXO Impacts Expression of NK Associated Genes Already at the CLP Stage

**Figure 5:**
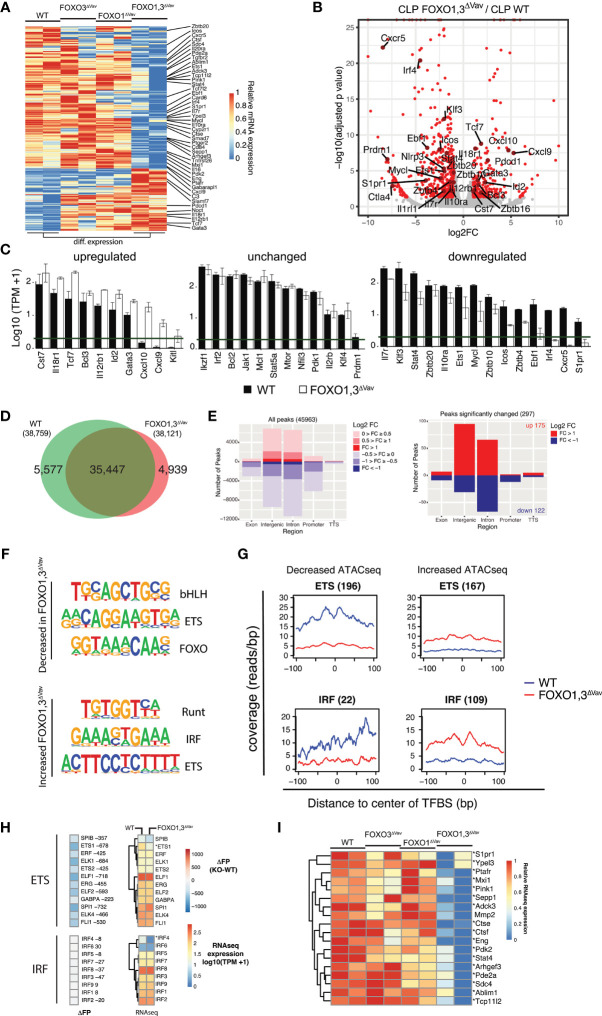
Removal of FOXO results in NK associated gene regulatory changes at the CLP stage. **(A)** Heatmap showing row normalized expression of genes with differential expression (adjusted p-value ≤0.01, ≥2-fold change in expression and ≥1 TPM in 2+ samples) between WT and FOXO1,3^ΔVav^ LY6D^neg^ CLPs. **(B)** Volcano plot showing log2 fold change and adjusted p-value for the comparison of WT to FOXO1,3^ΔVav^ LY6D^neg^ CLPs. Red dots indicate genes with >2-fold change in expression and adjusted p-value < 0.05. **(C)** Bar graphs showing expression (TPM) of select NK associated genes. Genes with adjusted p-value ≤ 0.01, ≥2-fold change in expression and ≥1 TPM in 2+ samples were considered to have the decreased or increased expression. The green line indicates 1 TPM. **(D)** Venn diagram showing the overlap between ATACseq peaks identified in LY6D^neg^ CLPs from WT and FOXO1,3^ΔVav^ mice. Only peaks identified in ≥2 replicas each with >30 reads were considered. **(E)** Annotation of all ATAC-seq peaks identified (left) and peaks with significantly altered chromatin accessibility (adjusted p-value < 0.01 and ≥2-fold change in signal) when comparing LY6D^neg^ CLPs from WT and FOXO1,3^ΔVav^ mice (right). Number of regions and log2 fold change (FOXO1,3^ΔVav^/WT) in ATAC-seq signal is indicated. **(F)** Motifs enriched in differential ATAC-seq peaks. Top three most significantly enriched motifs existing in >10% of regions are displayed. **(G)** Cut-profiles for differential ATAC-seq peaks with ETS- and IRF-family transcription factor binding sites (TFBS). Number of regions with each TFBS is indicated in parenthesis. **(H)** Genome-wide difference in the number of footprints (identified in WT and FOXO1,3^ΔVav^ LY6D^neg^ CLPs) (left) and expression (right) of indicated genes from the ETS- and IRF-families. * indicates significant differences in gene expression between LY6D^neg^ CLPs from WT and FOXO1,3^ΔVav^ mice. **(I)** Expression of known ETS1 targets in LY6D^neg^ CLPs from mice with indicated genotypes.

With the loss of FOXO perturbing the NK developmental pathway already at the level of the CLP (**Figures 5A, B**), we next sought to investigate if we could identify NK lineage related changes at this step of development. To this end, we performed RNA-seq on LY6D^neg^ CLP (GR1/MAC1/NK1.1/TER119/CD3ε^-^CD11C^-^LY6C^-^IL-7Rα^+^FLT3^+^KIT^low^LY6D^-^) from WT, FOXO1^ΔVav^ mice, FOXO3^ΔVav^ mice, and FOXO1,3^ΔVav^ mice. We found 469 genes that displayed significant differential expression between LY6D^neg^ CLPs from FOXO1,3^ΔVav^ and WT mice **(** **Figures 5A, B** and **Table S2** **)**. The observed perturbation in gene expression became progressively more distinct with the loss of both FOXO1 and FOXO3 function **(** **Figure 5A** **)**. This suggests that the FOXO proteins have mainly synergistic functions at this step of development.

In agreement with earlier reports, we found that the *Il7ra* gene was significantly down-regulated **(** **Figures 5A–C** **)** confirming the known positive regulatory role of FOXO in controlling IL-7R*a* expression (9, 16). Further, looking specifically at genes previously described to be NK cell signature genes (28), we found that a significant number of these genes displayed altered expression at the LY6D^neg^ CLP stage **(** **Figures 5B, C** **).** Of note, we found that *Tcf7, Id2, Il18r1*, *Il12rβ1, Cxcl10*, and *Cxcl9* – all genes encoding proteins important for NK cell development, migration, and functions (31, 56–59) - were up-regulated in the FOXO1,3^ΔVav^ cells. Conversely, looking at the down-regulated genes, we interestingly found that *Ets1* – a gene known to be important for the development of NK progenitors and NK maturation (30, 60) - was significantly down-regulated in CLPs **(** **Figures 5A–C** **)**. The down-regulation of ETS1 was also confirmed at the protein level in CLPs **(** **Figure S6A** **).**

### The NK Associated Gene Regulatory Network in CLPs Is Perturbed by the Loss of FOXO

We next investigated whether the changes in the expression of known NK-cell-development genes could be related also to changes in the gene regulatory landscape. Using the assay for transposase-accessible chromatin followed by sequencing (ATAC-seq), we identified close to 46,000 open chromatin regions across analyzed LY6D^neg^ CLP with the vast majority existing both in WT and FOXO1,3^ΔVav^ cells **(** **Figure 5D** **)**. Overall, most of the identified open chromatin regions were localized in intergenic and intronic region **(** **Figure 5E**, left**)**. Next, we identified regions with significant differences (adjusted p-value <0.01 and ≥2-fold change in signal amongst peaks identified in ≥2 samples and having >30 reads) in chromatin accessibility between WT and FOXO1,3^ΔVav^. This revealed 297 regions that were mainly localized in intergenic and intronic regions **(** **Figure 5E**, right**)**. Hence, this suggests that loss of FOXO in LY6D^neg^ CLPs results in relatively few but distinct changes to overall chromatin accessibility and that the major effect on gene regulation is *via* distal elements while promoters remain largely unaffected.

To identify transcription factors where altered binding could cause the alterations in chromatin accessibility, we performed *de novo* motif enrichment analysis on the peaks with altered chromatin accessibility. This revealed that peaks with decreased accessibility in the FOXO1,3^ΔVav^ LY6D^neg^ CLP were enriched for transcription factor binding (TFBS) sites related to the bHLH-, ETS- and, as expected, the FOXO family **(** **Figure 5F** top**)**. Correspondingly, RUNT, IRF, and ETS motifs were found in regions that gained chromatin accessibility **(** **Figure 5F** bottom**)**. To further corroborate that TF binding was altered, we analyzed the Tn5 integration sites (cut-profiles) around the putative TFBS for each of these TF-families. Out of the motifs identified **(** **Figure 5F** **)**, ETS and IRF produced clear cut-profiles **(** **Figures 5G** and **S6B** **).** This supports that altered ETS and IRF binding directly contribute to the changes in chromatin accessibility while suggesting that the other TFBS are either not used or that TF binding does not produce distinct cut-profiles on these analyzed regions.

Altered binding of a single transcription factor might not cause significant changes to the overall chromatin accessibility at the level of a whole chromatin region as defined by the ATAC-seq peaks. A complementary approach is footprinting analysis, which instead attempts to localize sudden decreases in the number of reads within an open chromatin region to identify individual TF bound regions (61). By means of footprint analysis, we assessed the changes in genome-wide binding of ETS and IRF. We found no major changes in the overall number of IRF associated footprints (**Figure 5H**). This suggests that increased IRF binding is specifically associated with peaks displaying increased chromatin accessibility in FOXO1,3^ΔVav^ CLPs (**Figures 5F, G**), while the observed decrease *Irf4* expression (**Figures 5C, H**) has no major impact on overall IRF-binding.

In contrast, we found a significant decrease in footprints associated with ETS motifs also at the genome-wide level **(** **Figure 5H** **)**. The fact that Ets1 is the only identified ETS-family member displaying significant changes in expression (**Figures 5C, H**), suggests that the decreased number of ETS-bound regions reflects reduced binding of ETS1. This conclusion is further supported by putative ETS1 target genes (62, 63) including *Stat4, Pdk2, Adck3* showing significantly lower expression in FOXO1,3^ΔVav^ CLPs **(** **Figure 5I** **)**. Taken together with the reduced expression of ETS1 (**Figures 5B, C** and **S6A**) and clear ETS cut-profile (**Figure 5G**), this suggests that loss of ETS1 binding in FOXO1,3^ΔVav^ CLPs contribute to the altered gene regulatory landscape and potentially the reduced capacity to generate NK cells downstream of the CLP (30).

We further looked specifically at TCF7 (31) and NFIL3 (29), as both are known to be critical for BM NK progenitor development. TCF7 expression was increased in CLPs from FOXO1,3^ΔVav^ mice **(** **Figures 5B, C** **)** but no significant change in binding as assessed by footprinting analysis (46 less footprints in the FOXO1,3^ΔVav^ CLPs) was observed. Hence, TCF7 is potentially controlled by FOXO at the transcriptional level but only has a minor impact in terms of chromatin accessibility at the CLP stage. NFIL3 showed no significant change in expression or overall binding (seven less footprints in the FOXO1,3^ΔVav^ CLPs). Hence, we find no indication that perturbed TCF7 and NFIL3 activity contribute to the changes observed in FOXO1,3^ΔVav^ CLPs.

### preNKPs Lacking FOXO Fail to Up-Regulate NK-Lineage Related Genes

**Figure 6:**
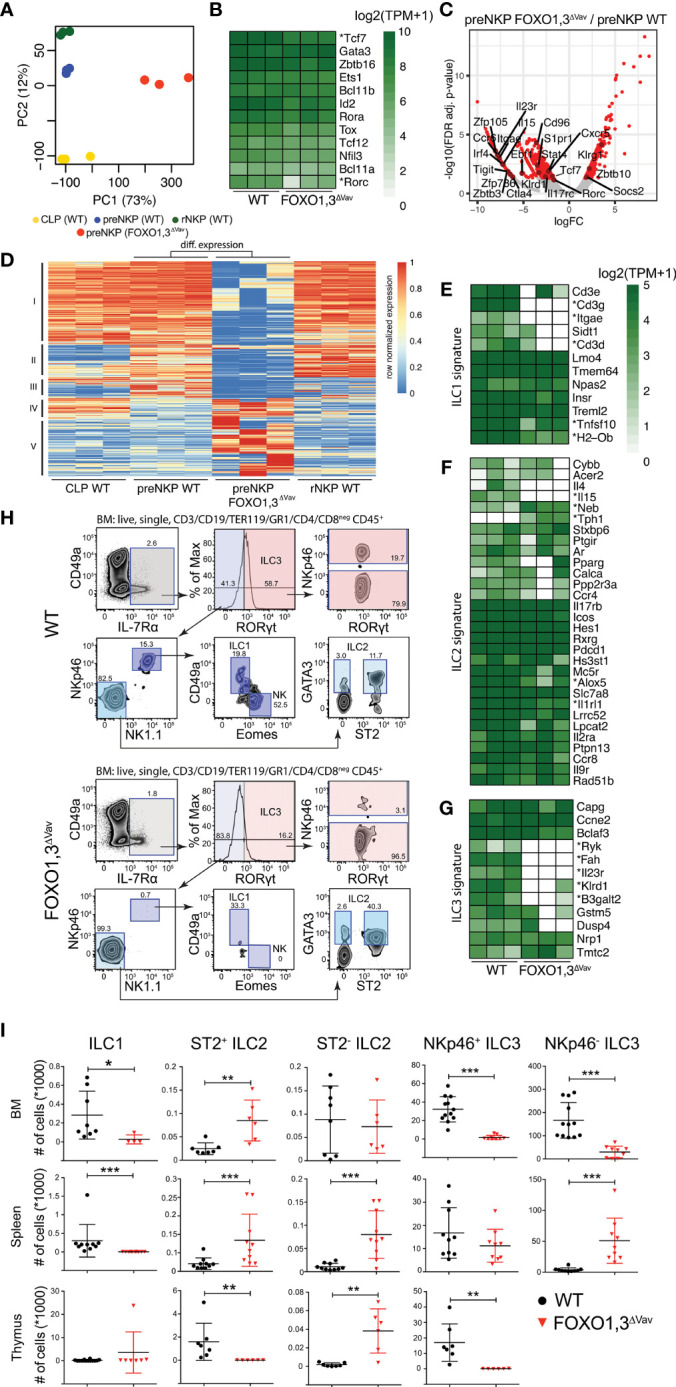
Loss of FOXO results in a perturbation of the preNKP transcriptional program and the development of non-cytotoxic ILCs. **(A)** Principal component (PC) analysis of RNAseq data from indicated cell populations (for gating strategy see **Figure 1A**) from wildtype and FOXO1,3^ΔVav^ mice (n = 3). The variation explained by each PC is displayed in parenthesis. **(B)** Hierarchically clustered heatmaps showing gene expression for selected transcription factors critical for NK/ILC development. * indicates significant differences in expression between WT and FOXO1,3^ΔVav^ preNKPs (FDR < 0.05, >2-fold change). **(C)** Volcano plot showing log2 fold change and adjusted p-value for the comparison of WT to FOXO1,3^ΔVav^ preNKPs. Red dots indicate genes with >2-fold change in expression and adjusted p-value < 0.05. **(D)** Hierarchically clustered heatmap showing row normalized expression of differential expressed genes (identified in C). Clusters I-V are indicated. **(E–G)** Hierarchically clustered heatmaps showing gene expression of **(E)** ILC1, **(F)** ILC2 and **(G)** ILC3 signature genes defined by Robinette et al. (64), * indicates significant differences in expression between WT and FOXO1,3^ΔVav^ preNKP (FDR < 0.05, >2-fold change). **(H)** Representative flow cytometry profiles showing the identification of ILC subsets in BM from WT and FOXO1,3^ΔVav^ mice. **(I)** Total number of indicated ILC subsets in BM, spleen and thymus from WT and FOXO1,3^ΔVav^ mice (n = 6-12). NKp46- ILC3 population had less than 50 cells per thymus in all mice from both mouse strains so was not shown. Dots represent individual analyzed animals. Bars indicate mean and SD. P-values were calculated using Mann-Whitney tests with *, ** and *** indicates p-values <0.05, <0.01 and <0.001 respectively.

With FOXO1,3^ΔVav^ mice displaying a block at the preNKP to rNKP transition, we next sought to characterize the transcriptional changes caused by the loss of FOXO in preNKPs. Based on PCA, the FOXO1,3^ΔVav^ preNKPs overall maintained a preNKP transcriptional profile as compared to its WT counterparts (PC2) with the top loadings of PC2 being CLP and NK-lineage related (**Figures 6A** and **S7A**). In line with this, the expression of Id2 – which marks the formation of preNKP from CLP (24) – was not altered and generally the expression of ILC related transcription factors (37) was also found to be similar **(** **Figure 6B** **)**. However, the combined loss of FOXO1 and FOXO3 did cause distinct gene expression changes as observed both by PCA (PC1) (**Figure 6A**) and direct comparison of expression profiles (**Figure 6C** and **Table S1**). In relation to normal development, the FOXO1,3^ΔVav^ preNKPs displayed lower expression of genes commonly expressed throughout the early NK progenitor hierarchy (**Figure 6D**, cluster I) and, to a lesser extent, maintained expression of CLP associated genes (**Figure 6D**, cluster IV). We additionally found that the FOXO1,3^ΔVav^ preNKPs failed to properly express genes normally upregulated in the CLP to preNKP transition and further increased in expression in the subsequent preNKP to rNKP transition (**Figure 6D**, cluster II). The later gene cluster was as expected enriched for NK lineage associated genes (**Figure S7B**) including amongst others *Klrd1* (encoding for CD94) and *Tigit* shown to function as NK immune checkpoint inhibitors (65). The reduced generation of rNKPs from FOXO1,3^ΔVav^ preNKPs is hence associated with a failure to properly express an early NK gene program.

Looking specifically at individual genes impacting NK development, we found that TCF7 - in contrast to what was observed in CLPs (**Figures 5B, C**) - was downregulated in FOXO1,3^ΔVav^ preNKPs while NFIL3 expression remained unaffected (**Figures 6B, C**). Hence, the lower TCF7 expression in preNKPs could potentially contribute to the impaired generation of rNKPs (31). Further, in line with data from the CLP stage, we found that ETS1 displayed a clear trend toward being down-regulated in FOXO1,3^ΔVav^ preNKPs (**Figures 6B** and **S7C**). In concordance with the CLP expression and epigenetic data, this argues for the loss of FOXO causing ETS1 downregulation which in turn contributes to the observed impairment generation of NK cells. Further in line with perturbed ETS1 expression contributing to the observed NK phenotype, we found that *Ets1* expression was significantly down-regulated also in splenic NK cells (**Figure S7D** **).**

Interestingly, we also found a significant decrease in expression of zinc finger protein 105 (*Zfp105)* **(** **Figure 5B** **)** – a transcription factor previously shown to be regulated by FOXO1 (15, 66), to augment differentiation toward NK cell lineage (67) and to be a direct target of FOXO1 in CD8 T cells – which might suggest that FOXO-mediated regulation of Zfp105 expression plays a role in NK cell development.

### Loss of FOXO1 and FOXO3 Disrupts Development of Non-Cytotoxic ILCs

The preNKP represents a heterogenous population which gives rise not only to NK cells but also to the other non-cytotoxic ILC subsets (24, 25). In agreement with this, we observed expression of genes encoding transcription factors linked to development of the non-cytotoxic ILCs, including *Tcf7, Tox, Bcl11b, Zbtb16, Rorc, Ets1, Nfil3* (37) in both WT and FOXO1,3^ΔVav^ preNKP **(** **Figure 6B** **)**.

We next sought to investigate if we could observe transcriptional changes in the FOXO1,3^ΔVav^ preNKP indicative of disruptions potentially influencing also the non-cytotoxic ILC subsets. To this end, we utilized the ILC1-3 gene signature published by Robinette et al. (64). Interestingly, we found down regulation of expression across all the three gene signatures (**Figures 6E–G**) but most notably within the ILC1 (**Figure 6E**) and ILC3 (**Figure 6G**) gene signatures.

These transcriptional changes made us speculate that the development of the non-cytotoxic ILC subsets in addition to NK cells could be perturbed in FOXO1,3^ΔVav^ mice. To investigate this, we performed phenotypic analysis of ILC subsets from BM, spleen, and thymus from WT and FOXO1,3^ΔVav^ mice (**Figure 6H**). Indeed, we found that the ILC1 population was reduced in BM and spleen (**Figures 6H, I**). ILC3 numbers were similarly reduced in BM and thymus while, in contrast, NKp46^-^ILC3s were increased in spleen (**Figures 6H, I**). In contrast, ILC2 subsets were generally increased in the analyzed organs (**Figures 6H, I**). Hence, we concluded that the loss of FOXO causes broad perturbation of the non-cytotoxic ILC subsets. Potentially these changes are due to gene regulatory changes already at the level of the preNKP, meaning that FOXO determine lineage specification of ILC.

## Discussion

In this study, we show that FOXO1 and FOXO3 are expressed in the early progenitors of the innate lymphoid lineages and cooperatively regulate the generation of NK cell progenitors and NK cells. In addition, we discovered a hitherto undescribed role of the FOXO family in establishing the NK/ILC gene expression program in progenitor cells and in the development of the ILC1, 2, and 3 subsets. Hence, the loss of FOXO1 and FOXO3 disrupts development of both the cytotoxic and non-cytotoxic ILC lineages.

Using a combination of RNA-seq and ATAC-seq data to study the underlying gene regulatory mechanisms, we found that the loss of FOXO proteins disrupted the regulation of NK and ILC associated genes already at the CLP stage and more markedly so at the preNKP stage. Likely the failure to establish the NK/ILC gene program in preNKPs directly results in the observed reduction in rNKPs. Interestingly, we found a decrease in ETS1 at the CLP stage onwards in FOXO1,3^ΔVav^ mice. In line with ETS1 being a critical downstream target of FOXO, the NK cell phenotypes of the ETS1 knockout very much resemble the FOXO1,3^ΔVav^ phenotype with reduced splenic NK cells and rNKPs while preNKPs seemingly remain unaffected (30, 60). Further, altered NKp46 and Ly49D expression were also observed in ETS1 deficient animals (30). With the activity of ETS1 being modulated *via* interaction with FOXO1 (68), the loss of FOXO could mimic the ETS1 knockout by both lowering ETS1 expression and ETS1 activity throughout NK cell development.

The preNKP compartment is heterogeneous and contains several progenitor populations out of which only a subset is involved in the generation of the NK lineage (24, 25). While these subsets are yet to be readily identifiable without the use of reporter genes, we observed distinct changes both in the activation of the overall preNKP gene expression program and in genes associated with the ILC1-3 lineages. These changes were associated with decreased numbers of ILC1 and ILC3 as well as increased numbers of ILC2. Hence, this argues that the altered gene expression caused by the loss of FOXO is directly reflected in the ILC lineages though it is unclear if these alterations reflect changes at the progenitor composition of the preNKP compartment or in the overall transcriptional program. Addressing this point will require further studies using reporter mice to distinguish the different progenitor populations within the preNKP compartment.

The loss of FOXO could also directly influence the development of ILCs (i.e., by acting downstream of important preNKP transcription factors). Indeed, the expression of BCL11B in preNKP, especially in combination with ZBTB16, marks the development of ILC2 (24). On the other hand, the absence of both markers in preNKP allows for a balanced development of NK cells and all ILC lineages (24). FOXO proteins have been suggested to repress cell cycle progression downstream of BCL11B, which is a critical regulator of basal cell quiescence in the mammary gland (69). ZBTB16 overexpression leads to reduced FOXO phosphorylation (70) while FOXO1 expression is induced in ZBTB16 heterozygosity as compared to homozygosity (71). Altogether, this makes it tempting to speculate that FOXO serve as crucial transcriptional regulator acting downstream of ZBTB16 and/or BCL11B to control ILC development in a cell type-specific manner.

Prior studies using Ncr1-iCre and Vav-iCre have reported contradictory data on the role of the FOXO1 in NK cell development (32–34). Using the VAV-iCre model, we in agreement with Deng et al., found that the deletion of FOXO1 did not significantly alter total NK cell numbers while CD27^low^CD11b^high^ NK cells were accumulated in line with FOXO1 suppressing Tbx21 expression needed for maturation (32). However, in contrast to what was observed following the combined deletion of FOXO1 and FOXO3 in the Ncr1-Cre model, we found that NK cell numbers declined significantly in both the BM and spleen of FOXO1,3^ΔVav^ animals. The splenic NK cells that developed displayed an accumulation of immature CD11b^low^ NK cells but lacked the accumulation of CD27^low^CD11b^high^ NK cells observed after the deletion of FOXO1 alone. The discrepancy in maturation status between FOXO1,3^ΔNcr1^ and FOXO1,3^ΔVav^ might be attributed to their differential roles in hematopoietic progenitors and committed NK cells.

In addition, FOXO1 single deficient mice exhibit an increase in terminally mature splenic NK cells, suggesting that FOXO1 exerts negative regulation on NK cell maturation. These data support the model where FOXO1 inhibit NK cell maturation by repressing Tbx21 (32). The additional deletion of FOXO3 reversed this phenotype leading to accumulation of immature subsets potentially suggesting that in the periphery FOXO1 might act as a brake for FOXO3 driven maturation. Thus, both FOXO1 and FOXO3 expression is key rheostat of NK cell maturation. Such a reciprocal regulation was recently suggested for Tbx21 and Eomes in NK cell maturation (72). FOXO1 acted also as a negative regulator for NK cell receptors DNAM-1 and KLRG1. At least for the DNAM-1 receptor, this might be independent of the maturation status, as we found that immature and mature NK cell subsets were similarly represented in DNAM-1^−^ and DNAM-1^+^ NK cells (73).

Somewhat surprisingly, BM NK cell maturation in the FOXO1,3^ΔVav^ animals was left rather unperturbed. Potentially, this argues for the lower NK cell numbers in BM being a consequence of the reduced numbers of rNKPs rather than major issues with NK cell maturation. Speculatively, this would in addition suggest that NK maturation in spleen and BM to some extent have different requirements and that the environment causes different reliance on the FOXO proteins.

IL-15 signaling is critical for NK cell development and survival (74–76). We found that the FOXO1,3^ΔVav^ NK cells displayed markedly reduced CD122 (IL15Rβ) and that expression was quantitatively correlated with NK cell numbers. Supporting the notion that the FOXO1,3^ΔVav^ NK cells have reduced IL-15-signaling, we also found that the IL-15 dependent expression of NKG2D (53–55) was significantly lower on BM and splenic NK. Hence, this argues that the reduced NK cell numbers in FOXO1,3^ΔVav^ mice result from defects in the generation of NK cells *via* rNKPs in combination with decreased ability to respond to IL-15 signaling critical for maintaining the normal NK population. Furthermore, the reduction of the ILC1 population in the absence of FOXO1,3 could be explained to occur similarly to the reduction of the NK cell population through their indispensable requirement of IL-15 needed for cell development (38, 77).

It is appealing to speculate that the changes in progenitor development as well as the reduced CD122 expression can be attributed to the reduction in ETS1 expression. Indeed, chromatin immune precipitation experiments revealed that ETS1 binds to the promoter of the CD122 gene and CD122 expression was reduced in mature ETS1 KO NK cells (30). FOXO1 and FOXO3 hence might tune CD122 expression indirectly through regulating a network of factors including ETS1 to gradually modulate IL-15 responsiveness, rather than causing an “on/off” situation in CD122 expression. Given that once NK cells acquire NKp46 expression (and hence gene deletion occurs in the Ncr1-Cre model), CD122 expression is not perturbed by the loss of FOXO1 and FOXO3 activity (32), this suggests that the loss of FOXO activity in FOXO1,3^ΔVav^ NK progenitors or very early NK cells (prior to Ncr1-Cre mediated deletion) cause a defect that cannot be corrected in later stages of NK cell development. This hypothesis is in line with recent studies showing that IL-15 signaling creates a positive regulatory loop to modulate expression of its receptors and several components of the IL-15 signaling pathway (40). Hence, FOXO1 and FOXO3 would then serve as crucial early regulator of NK cell fate by establishing proper IL-15 receptor expression.

In conclusion, the co-expression and regulatory function of FOXO1 and FOXO3 is critical throughout the NK cell development and maturation. Mechanistically, we propose that FOXO1 and FOXO3 – amongst other genes - control the expression of ETS1 and CD122 that both are integral for NK cell development. In addition, FOXO proteins selectively promote the development ILC1 and ILC3 but not ILC2. The very well controlled intrinsic modes of NK cell development, differentiation, and maturation by FOXO1 and FOXO3 revealed in our study can drive future efforts to develop anti-tumor and anti-viral immunotherapies targeting FOXO proteins.

## Materials and Methods

### Mice

To generate animals conditionally lacking FOXO1 and/or FOXO3 throughout the hematopoietic system, we crossed *FOXO1^flox/flox^* (47) and/or *FOXO3^flox/flox^* (48) with Vav^-^iCre (46) mice. All alleles were maintained on a C57BL/6 background and mice were predominantly analyzed at 8 to 14 weeks of age. Congenic CD45.1 WT C57BL/6 mice were used as recipients in transplantation experiments. All animal experiments were approved by the local animal ethics committee.

### Flow Cytometry

Single-cell suspension of bone marrow, spleen, blood, or thymus were incubated with Fc block (anti-FcγRIII, clone 2.4G2) and subsequently stained with fluorescent antibodies (**Table S3**) and viability markers (LIVE/DEAD^®^ Fixable Aqua Dead Cell Stain Kit or propidium iodide, both from Invitrogen). Staining was done at 4°C in PBS with 2% FBS for 20 min. Results were acquired mainly using the BD LSRFortessa™ or BD FACSymphony™ Flow Cytometers (BD Biosciences).

For FACS sorting of progenitor cells, mature cells were depleted using antibodies against TER119, CD3, CD19, GR1, and MAC1 together with sheep anti-rat IgG Dynabeads (Invitrogen) prior to staining with fluorescent antibodies. Cell sorting was performed mainly on a FACSAriaIIu or FACSAria Fusion (BD Biosciences).

Further analysis of FACS data was performed using Flowjo v9.9.6 (TreeStar, Ashland, OR). Corrected mean fluorescence intensities (MFI) were calculated by subtracting the MFI of control sample (stained only with secondary antibody) from the MFI of the FOXO stained sample. Normalized MFIs were calculated by dividing MFIs with the average MFI observed for WT samples in each independent experiment.

### Transplantation Assay

To determine *in vivo* NK lineage output from FOXO1,3^ΔVav^ hematopoietic stem- and progenitor cells, unfractionated bone marrow (0.2 x 10^6^ WT or 3 x 10^6^ FOXO1,3^ΔVav^ CD45.2 donor cells), was injected intravenously into irradiated (950cGy) CD45.1 recipients. The number of unfractionated BM cells transplanted were proportional to the frequency of phenotypic hematopoietic stem cells in WT and FOXO1,3^ΔVav^ mice (data not shown). Part of the FOXO1,3^ΔVav^ transplanted animals were in addition given 0.2 x 10^6^ unfractionated WT BM cells as support. Reconstitution was analyzed at 12 weeks post-transplantation using flow cytometry.

### TotalScript Based RNA-Seq and Analysis

RNA-seq was done using TotalScript (Epicenter) on RNA prepared from approximately 5000 FACS sorted cells using RNeasy micro (Qiagen) as previously described (78). Libraries were sequenced pair end (2x50 cycles) on the Illumina platform. Reads were mapped to the mouse reference genome (mm10) using STAR v2.3.2b (https://github.com/alexdobin/STAR). Strand-specific reads in exons were quantified using HOMER and assessment of differential gene expression analysis was done using EdgeR (79) on raw read count. Data visualizations were mainly done using ggplot2, pheatmaps, and R base graphics.

### ATAC Sequencing and Analysis

ATAC sequencing was performed (using 3000-5000 FACS sorted cells) as previously described (80). Libraries were sequenced pair-end (2x50 cycles) on the Illumina platform (Illumina). Reads were trimmed (using Trim Galore v0.4.1), mapped to the mouse reference genome (mm10) (using Bowtie2 v2.3.3.1) and PCR duplicates removed when making HOMER tag directories (using makeTagDirectory with -tbp 1). Peaks were subsequently identified in sub-nucleosomal reads (read-pairs within 100bp) using HOMER’s findPeaks.pl. Peaks with differential chromatin accessibility were identified using EdgeR on raw read counts in identified peaks. Peaks displaying an adjusted p-value ≤0.01 and ≥2-fold change in read count were considered to have differential chromatin accessibility. Only peaks identified in ≥2 replicas each with >30 reads were considered in the analysis. Annotation and motif enrichment analysis of differential peaks were done using the HOMER’s annotatePeaks.pl and findMotifsGenome.pl with -size given respectively.

To make cut-profiles, the localization of known HOMER transcription factor binding sites (TFBS) belonging to members of the enriched TF family (identified by the motif enrichment in differential ATAC-seq peaks) were localized in the genome using HOMER’s findMotifsGenome.pl. To take into account the position of the Tn5 integration into the genome, custom HOMER tag directories were made off-setting reads on the plus and minus strand with +4 and -5 bases respectively. Read depth centered around TFBS from a specific family were subsequently plotted using HOMER’s annotatePeaks.pl with a -fragLength of 9 (corresponding to the bp covered by Tn5) and -hist 1 (1 bp bins).

Genome-wide footprinting to identify TF binding sites was performed using DNase2TF. In brief, Bowtie2 mapped reads were deduplicated (using Picacard tools’ MarkDuplicates), data from the same population/genotype merged (using SamTools’ merge) and down-sampled to 39 million read-pairs per sample (using Picard Tool’s DownsampleSam.jar). Localization of reads were off set in the.bam file to take into account the Tn5 integration (as described above) using custom scripts. HOMER tag directories and peak finding were done as described above. Peak files and downsampled.bam files were subsequently used as input for DNase2TF (81). Identified footprints with a p-value ≤0.05 were overlapped with the TFBS identified in the mouse reference genome (mm10) using the transfac catalogue as previously described (82). Footprints were associated with TFBS when the center of the TFBS fell within the footprint.

Data visualizations were mainly done using ggplot2, pheatmaps and R base graphics.

### RTqPCR

NK cells were FACS sorted using BD AriaIII (BD Biosciences). CD117+ BM cells were enriched using CD117 MicroBeads, mouse (Miltenyi). RNA was purified using RNeasy micro kit (Qiagen) and cDNA prepared using MultiScribe Reverse Transcriptase (Life Technologies) or SuperScript II (Life Technologies) in combination with random hexamer priming. qPCR was performed using TaqMan™ Universal PCR Master Mix (Life Technologies) and TaqMan probes against: FOXO1 (Mm00490672_m1), FOXO3 (Mm00490673_m1), hprt (Mm01545399_m1 or Mm00446968_m1) and Ets1 (Mm01175819_m1).

### SMART-Seq Based RNA-Seq and Analysis

Two hundred to 500 progenitor cells were FACS sorted into lysis solution with DNase I from the Single Cell Lysis Kit (Invitrogen) and samples prepared according to manufacturer’s instructions. RNA-seq libraries were subsequently prepared using the SMART-Seq Stranded Kit (Takara) according to the manufacturer’s instruction. Quality of cDNA library was determined using an Agilent Bioanalyzer according to the manufacturer’s protocol. Libraries were quantified by using the KAPA-SYBR FAST qPCR kit (Roche) and sequenced pair-end (2x75 cycles) on the Illumina NextSeq 500.

Adaptor sequences were trimmed, and low-quality reads removed using Trimmomatic (v.0.36). All sequencing reads aligning (HiSAT2, v.2.1.0) to annotated mouse ribosomal RNA genes were discarded. High-quality and ribosomal RNA depleted sequencing reads were aligned to the genome GRCm38.p6/mm10 genome using HiSAT2. Using sorted bam files (Samtools v.1.10), the number of aligned reads were counted (featurecount in subread package v. 2.0.0). After normalization (TMM: trimmed mean of M-values), a differential gene expression analysis (edgeR v. 3.28.1) was performed. Significant differentially expressed genes were distinguished by a false discovery rate (FDR) <0.05. Data were plotted using ggplot2 (v.2.3.3) in R (v. 3.6.1). Gene ontology analysis was conducted using clusterprofiler (v.3.14.3) with the database org.Mm.eg.db (v.3.10.0) in R. All scripts used for processing of SMART-seq data are deposited on Github: https://github.com/jonasns/NK_FOXO.

### Statistics

Statistical analysis of FACS data was performed using Graphpad Prism version 6 for Mac OSX (Graphpad 83) or R version 3.3.3 (R Development Core 84). Statistics pertaining to RNA-seq and ATAC-seq data were performed as described above.

## Data Availability Statement

SMART-seq RNAseq data are deposited at https://www.ebi.ac.uk/arrayexpress/experiments/E-MTAB-9560/. RNA-seq and ATAC-seq data are available from the European Nucleotide Archive (www.ebi.ac.uk/en) under accession numbers PRJEB20316, PRJEB41018 and PRJEB40258.

## Ethics Statement

The animal study was reviewed and approved by Stockholms Södra Djurförsöksetiska Nämnd and Linköpings Djurförsöksetiska Nämnd.

## Author Contributions

TL performed experiments, analyzed data, and contributed to writing the manuscript. JS performed RNA-seq experiments, analyzed RNA-seq data and contributed to writing the manuscript. LP-P analyzed RNA-seq data, analyzed ATAC-seq data and contributed to writing the manuscript. SK FACS sorted cells and assisted with animal experiments. AK and CG performed RNA-seq and ATAC-seq experiments. SM and LS assisted with flow cytometry staining, *in vitro* experiments, and manuscript discussion. NF, YH, and TB assisted with animal experiments. MK provided critical input on the ATAC-seq analysis. AW and BC helped with functional assays and manuscript discussion. AA and CK assisted with RNA-seq experiments, bioinformatic analysis and discussion of the manuscript. PH, RM, and NK supervised the study. RM and NK designed the study, performed experiments, analyzed data, and wrote the manuscript. All authors contributed to the article and approved the submitted version.

## Funding

This work was supported by grants from The Swedish Cancer Society, Swedish Research Council, King Gustav V Jubilee Fund, The Karolinska Institutet Foundations, The Stockholm County Council, The Swedish Foundation for Strategic Research, The Knut and Alice Wallenberg Foundation and generously, a donation by Björn and Lena Ulvaeus. In addition, the Karolinska Institutet doctoral education program (KID) supported the doctoral studies of TL and LP-P.

## Conflict of Interest

The authors declare that the research was conducted in the absence of any commercial or financial relationships that could be construed as a potential conflict of interest.

## Publisher’s Note

All claims expressed in this article are solely those of the authors and do not necessarily represent those of their affiliated organizations, or those of the publisher, the editors and the reviewers. Any product that may be evaluated in this article, or claim that may be made by its manufacturer, is not guaranteed or endorsed by the publisher.
