# Supplementary material for: FOXO1 and FOXO3 Cooperatively Regulate Innate Lymphoid Cell Development
Source: Front Immunol. 2022 Jun 9;13:854312. doi: 10.3389/fimmu.2022.854312 (PMC9218573; doi:10.3389/fimmu.2022.854312)
Supplement: Supplementary file 1 [file DataSheet_1.pdf]

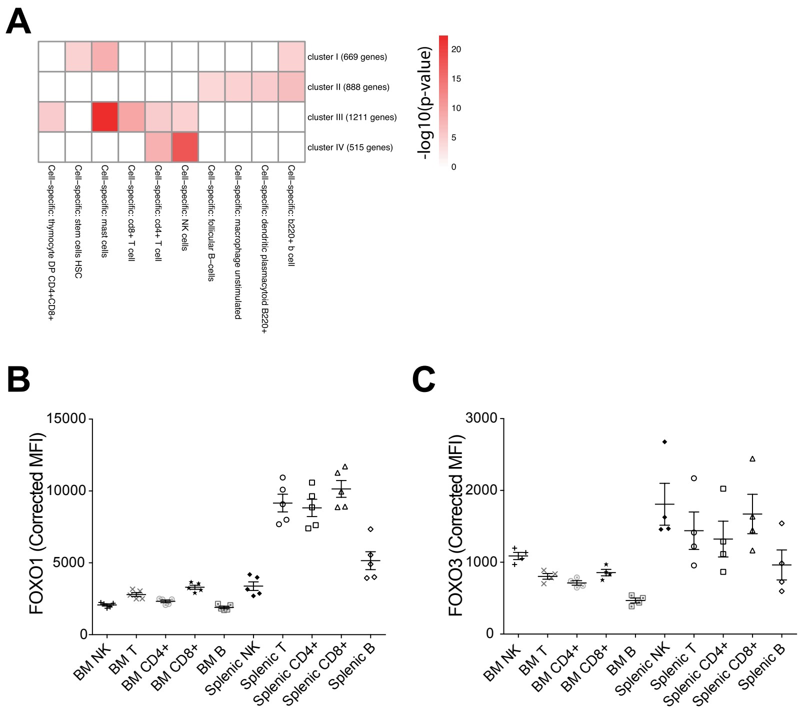

Fig. S1. Annotation of developmentally regulated genes and comparison of FOXO expression. (A) Metascape gene enrichment analysis on the gene clusters from Figure 1F. (B-C) FOXO1 (B) and FOXO3 (C) expression in NK cell subsets, T cell subsets, and B cells from C57Bl6 mice as measured by FACS in bone marrow and spleen samples. Dots indicate individual mice. Data is from one representative out of two experiments (*n* = 4-5). Bars indicate mean and SD.

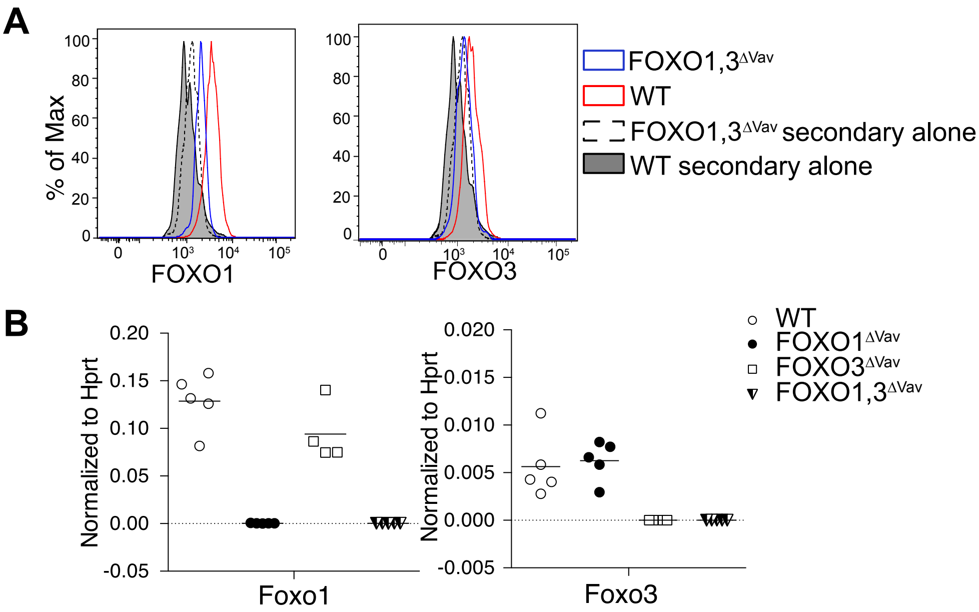

Fig. S2. Vav-iCre mediates effective deletion of the loxP flanked FOXO1 and FOXO3 regions. (A) FOXO1 and FOXO3 protein levels in splenic NK cells as measured by intracellular flow cytometry. (B) Expression of FOXO1 and FOXO3 mRNA in mice with the indicated genotype. Each dot represents data from an individual mouse. mRNA levels were measured in KIT (CD117) enriched bone marrow progenitors using RT-qPCR with probes specific to the loxP flanked regions coding for the DNA binding domains of FOXO1 and FOXO3 respectively.

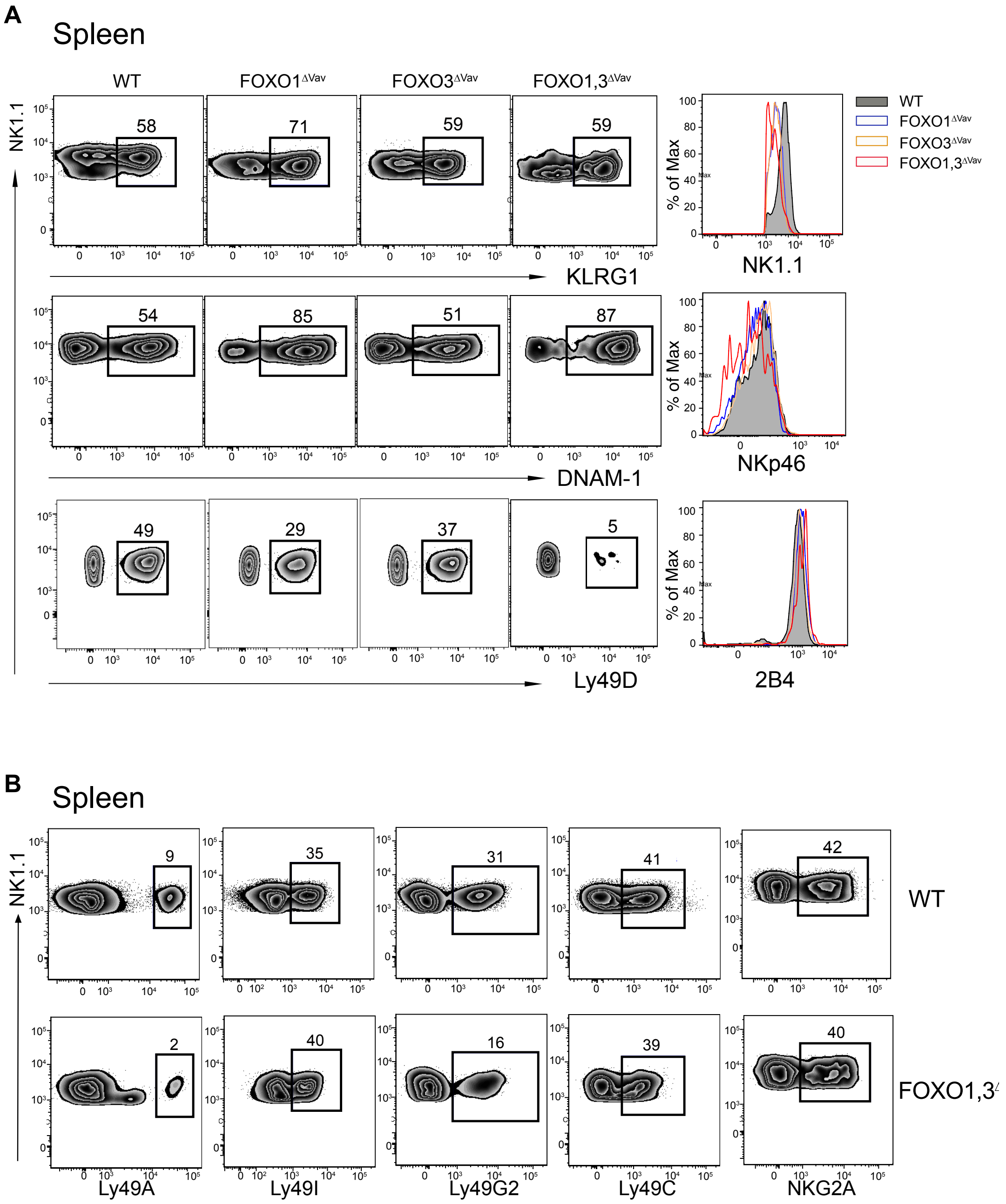

Fig. S3. Splenic NK cells from FOXO1,3$^{\Delta Vav}$ have perturbed activating and inhibitory receptor expression. Representative flow cytometry plots showing activating (A) and inhibitory (B) receptor expression on splenic NK cells.

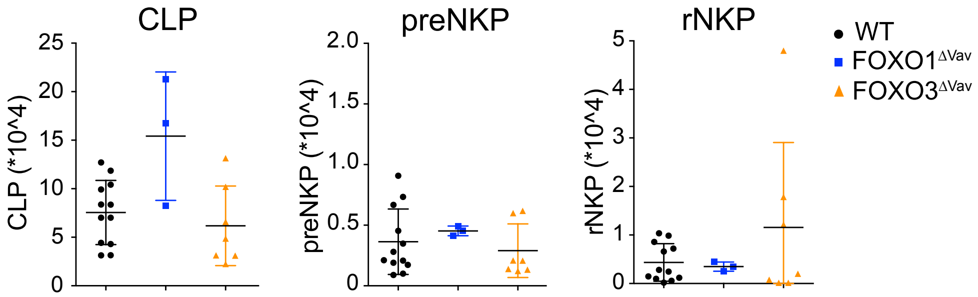

Fig. S4. Loss of FOXO1 or FOXO3 alone does not impact preNKP and rNKP numbers. Total number of CLP, preNKP and rNKP in BM of animals with the indicated genotype. Dots represent individual analyzed animals; p-values were calculated using Mann-Whitney; bars indicate mean and SD; *, ** and ** indicate p-values <0.05, <0.01 and <0.001 respectively.

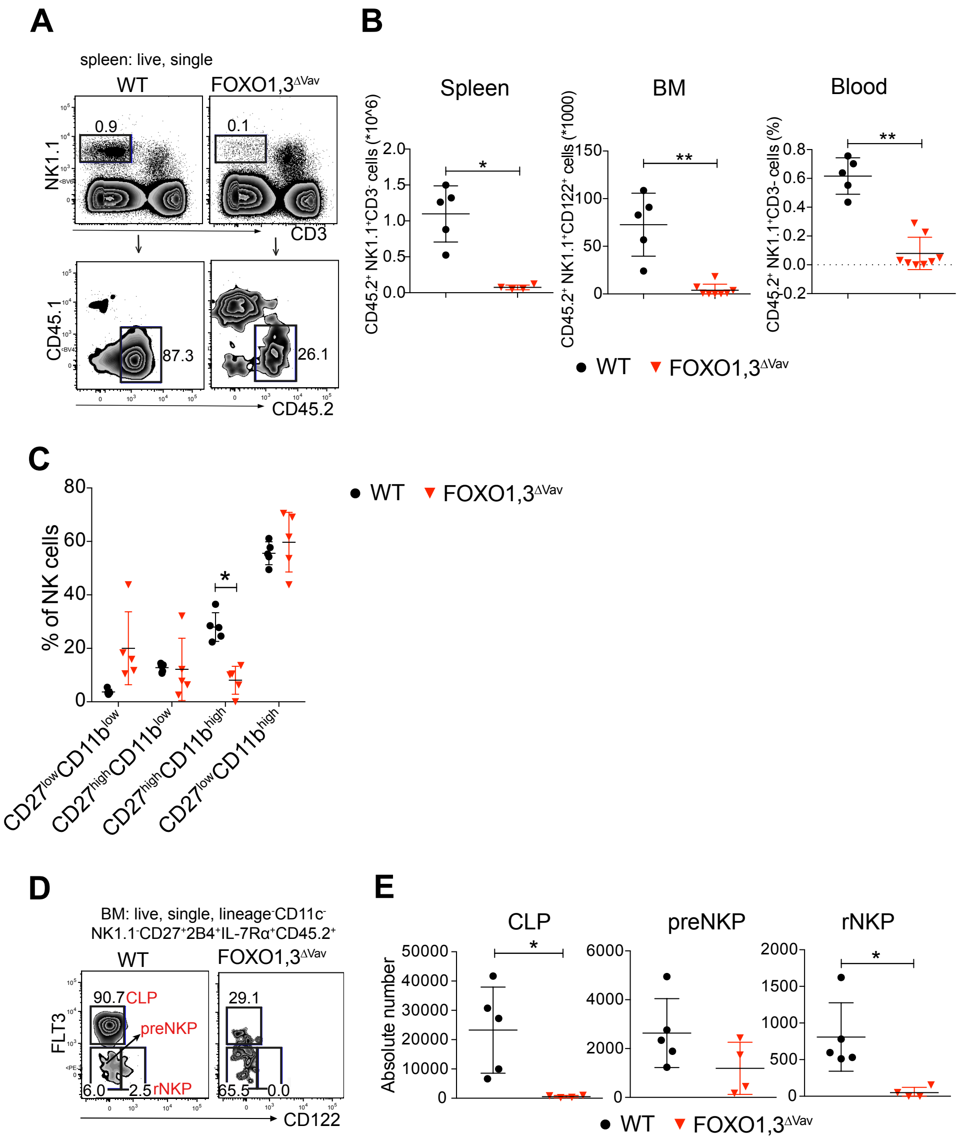

Fig. S5. The NK defect in FOXO1,3$^{\Delta Vav}$ mice is intrinsic to hematopoiesis. (A) Representative flow cytometry profile showing NK cell reconstitution in spleen 12 weeks post-transplantation. CD45.1 recipient mice were irradiated and infused with unfractionated BM cells from CD45.2 donors with the indicated genotype. Reconstituted NK cells were defined as NK1.1^{+}CD3^{+}CD45.2^{+}CD45.1^{-} cells. (B) Total number (in spleen and BM) and percentage (in blood) of donor NK cells 12 weeks post-transplantation (*n* = 4-8). (C) Frequency of splenic CD45.2+ NK cells at indicated maturation stages 12 weeks post-transplantation (*n* = 4-5). (D) Representative flow cytometry profiles showing the identification of CD45.2^{+} (donor) CLP, preNKP and rNKP in the transplanted animals. (E) Total number of CLP, preNKP, and rNKP in the transplanted animals (*n* = 4-5). In panels B, C and E: dots represent individual analyzed animals; p-values were calculated using Mann-Whitney; bars indicate mean and SD; *, ** and ** indicate p-values <0.05, <0.01 and <0.001 respectively.

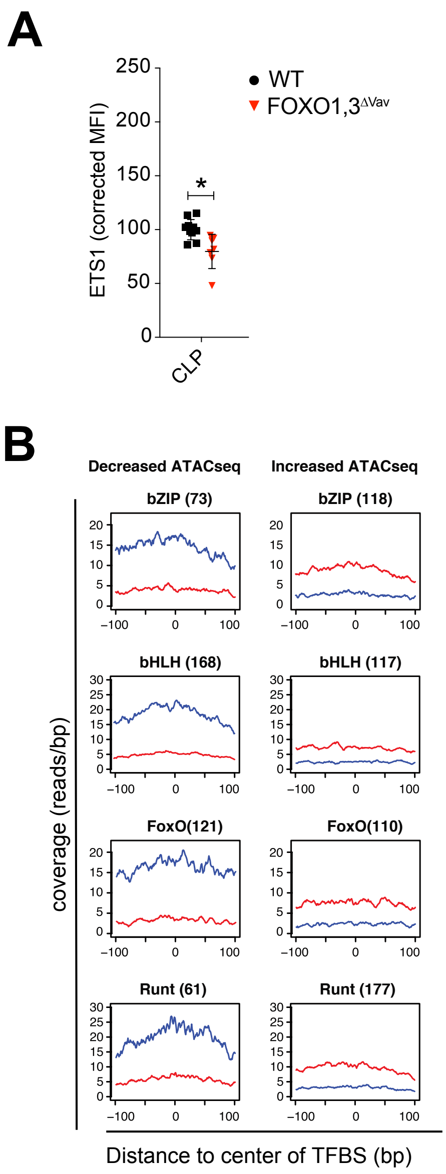

Fig. S6. ETS1 protein expression and transcription factor cut-profiles. (A) ETS1 expression at protein level as measured by intracellular flow-cytometry. Dots represent individual analyzed animals; p-values were calculated using Mann-Whitney-Wilcoxon test; bars indicate mean and SD; * indicate p-values <0.05. (B) Cut-profile of differential ATAC-seq peaks with bZIP, bHLH, FoxO and Runt transcription factor binding sites (TFBS). The number of TFBS found within the differential peaks is indicated in brackets.

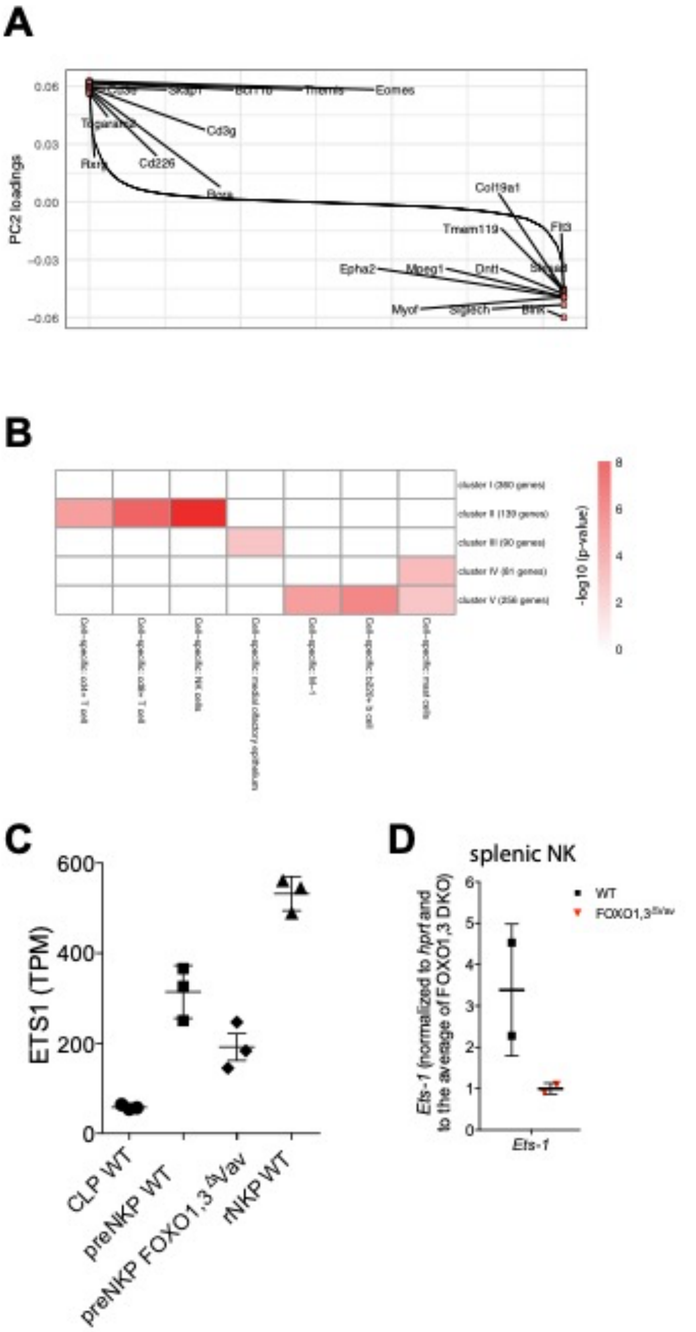

Fig. S7. Annotation of developmentally regulated genes and comparison of FOXO expression. (A) Top ten loadings contributing to PC2 in Fig. 6A. (B) Metascape gene enrichment analysis on the gene clusters from Figure 6D. (C) Ets1 expression (TPM) from RNAseq on CLP, preNKP and rNKP from indicated genotype. (D) RTqPCR of Ets1 and Tbx21 from WT and FOXO1,3$^{\Delta Vav}$ NK cells from spleen.
